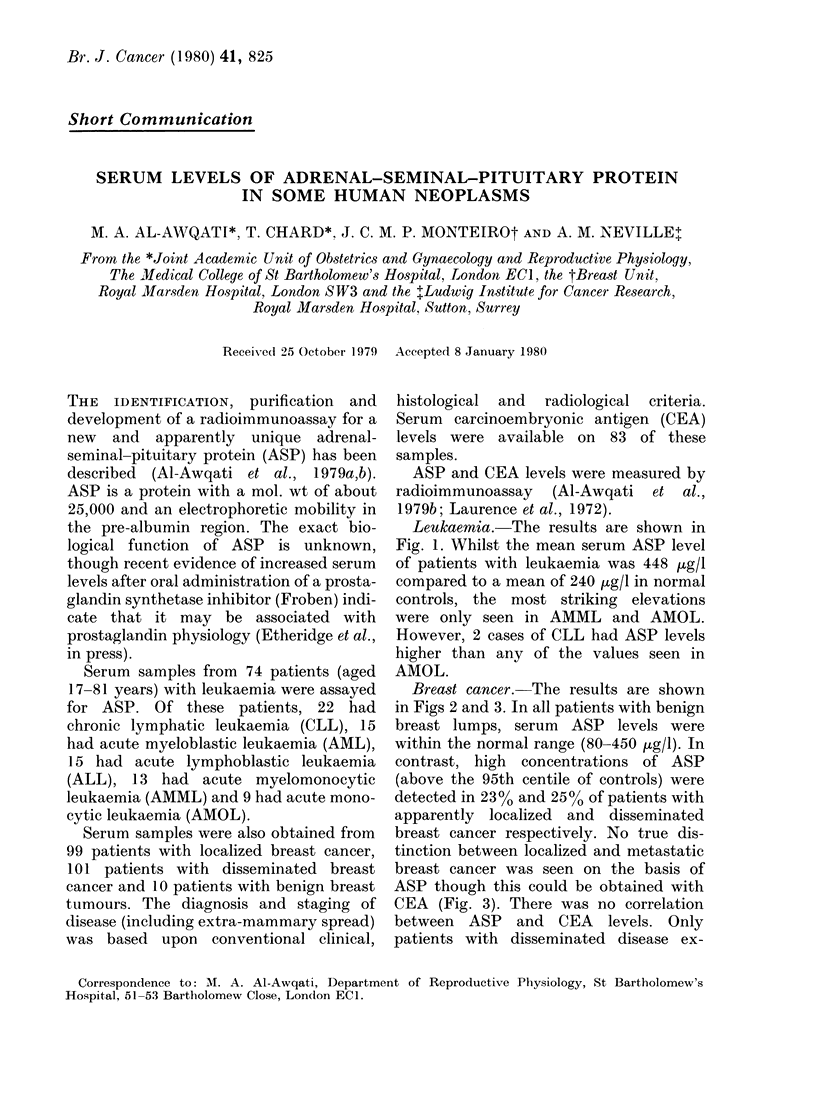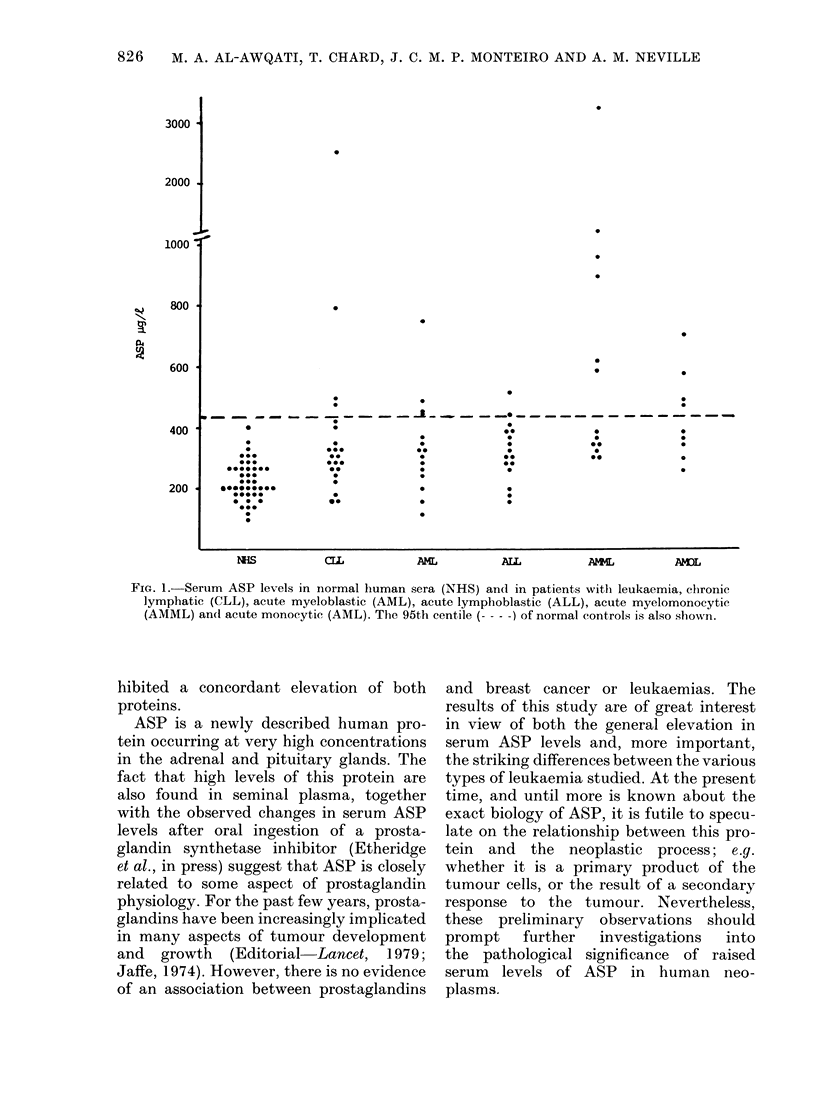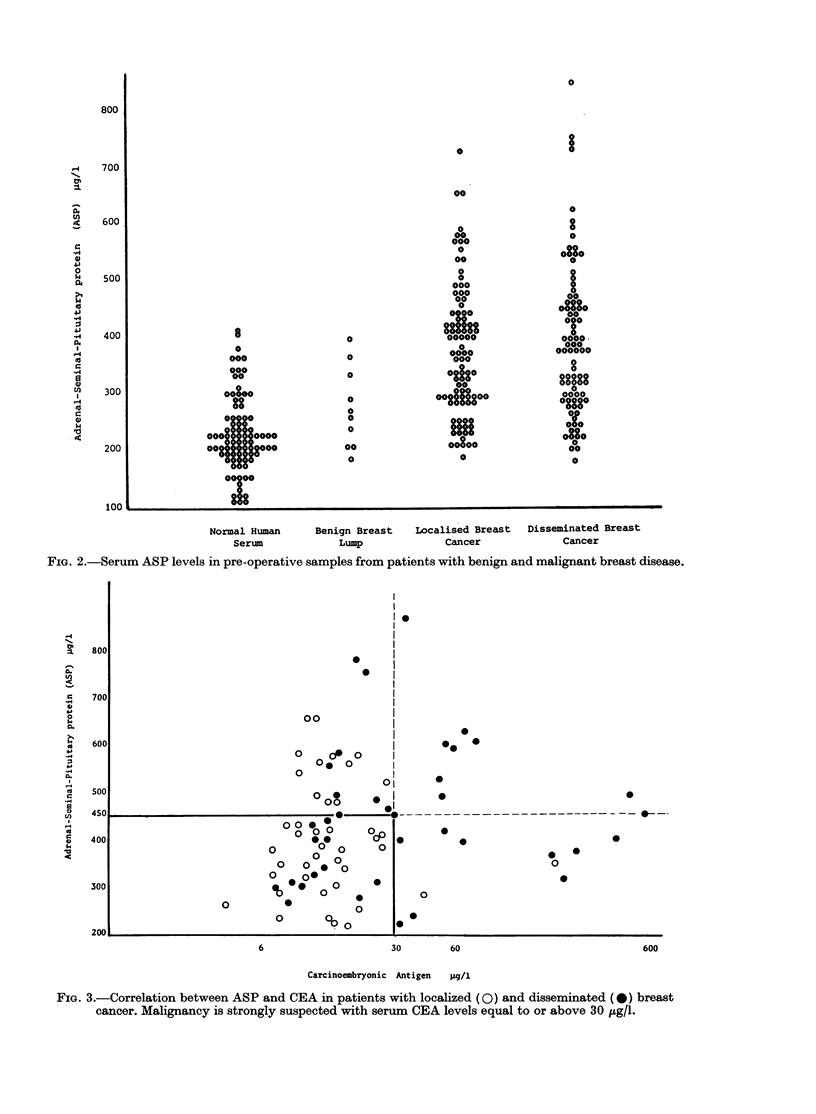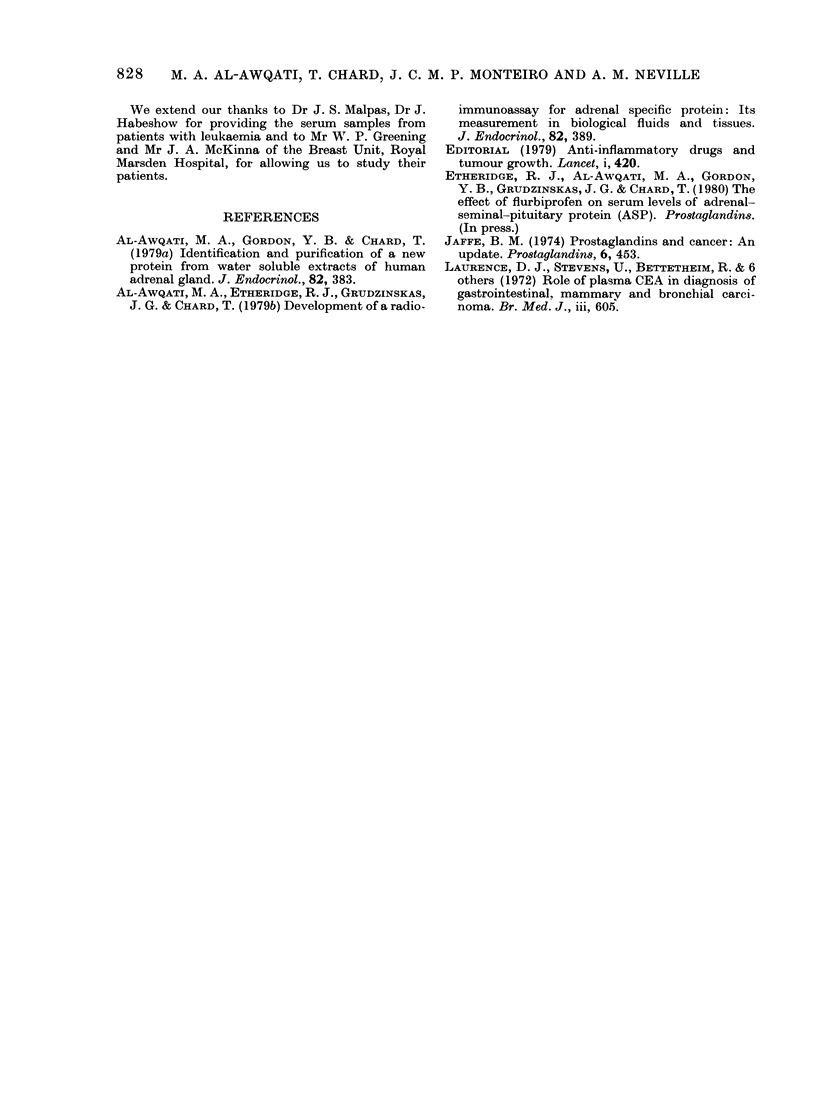# Serum levels of adrenal-seminal-pituitary protein in some human neoplasms.

**DOI:** 10.1038/bjc.1980.148

**Published:** 1980-05

**Authors:** M. A. Al-Awqati, T. Chard, J. C. Monteiro, A. M. Neville


*Br. J. Cancer* (1980) **41**, 825

***Short Communication***

### SERUM LEVELS OF ADRENAL–SEMINAL–PITUITARY PROTEIN IN SOME HUMAN NEOPLASMS

M. A. AL-AWQATI*, T. CHARD*, J. C. M. P. MONTEIRO† AND A. M. NEVILLE‡

*From the *Joint Academic Unit of Obstetrics and Gynaecology and Reproductive Physiology, The Medical College of St Bartholomew's Hospital, London EC1, the †Breast Unit, Royal Marsden Hospital, London SW3 and the ‡Ludwig Institute for Cancer Research, Royal Marsden Hospital, Sutton, Surrey*

Received 25 October 1979 Accepted 8 January 1980

THE IDENTIFICATION, purification and development of a radioimmunoassay for a new and apparently unique adrenal-seminal–pituitary protein (ASP) has been described (Al-Awqati *et al.*, 1979*a,b*). ASP is a protein with a mol. wt of about 25,000 and an electrophoretic mobility in the pre-albumin region. The exact biological function of ASP is unknown, though recent evidence of increased serum levels after oral administration of a prostaglandin synthetase inhibitor (Froben) indicate that it may be associated with prostaglandin physiology (Etheridge *et al.*, in press).

Serum samples from 74 patients (aged 17–81 years) with leukaemia were assayed for ASP. Of these patients, 22 had chronic lymphatic leukaemia (CLL), 15 had acute myeloblastic leukaemia (AML), 15 had acute lymphoblastic leukaemia (ALL), 13 had acute myelomonocytic leukaemia (AMML) and 9 had acute monocytic leukaemia (AMOL).

Serum samples were also obtained from 99 patients with localized breast cancer, 101 patients with disseminated breast cancer and 10 patients with benign breast tumours. The diagnosis and staging of disease (including extra-mammary spread) was based upon conventional clinical, histological and radiological criteria. Serum carcinoembryonic antigen (CEA) levels were available on 83 of these samples.

ASP and CEA levels were measured by radioimmunoassay (Al-Awqati *et al.*, 1979*b*; Laurence *et al.*, 1972).

*Leukaemia.*—The results are shown in Fig. 1. Whilst the mean serum ASP level of patients with leukaemia was 448 $\mu$g/l compared to a mean of 240 $\mu$g/l in normal controls, the most striking elevations were only seen in AMML and AMOL. However, 2 cases of CLL had ASP levels higher than any of the values seen in AMOL.

*Breast cancer.*—The results are shown in Figs 2 and 3. In all patients with benign breast lumps, serum ASP levels were within the normal range (80–450 $\mu$g/l). In contrast, high concentrations of ASP (above the 95th centile of controls) were detected in 23% and 25% of patients with apparently localized and disseminated breast cancer respectively. No true distinction between localized and metastatic breast cancer was seen on the basis of ASP though this could be obtained with CEA (Fig. 3). There was no correlation between ASP and CEA levels. Only patients with disseminated disease ex-

Correspondence to: M. A. Al-Awqati, Department of Reproductive Physiology, St Bartholomew's Hospital, 51–53 Bartholomew Close, London EC1.

FIG. 1.—Serum ASP levels in normal human sera (NHS) and in patients with leukaemia, chronic lymphatic (CLL), acute myeloblastic (AML), acute lymphoblastic (ALL), acute myelomonocytic (AMML) and acute monocytic (AML). The 95th centile (- - - -) of normal controls is also shown.

hibited a concordant elevation of both proteins.

ASP is a newly described human protein occurring at very high concentrations in the adrenal and pituitary glands. The fact that high levels of this protein are also found in seminal plasma, together with the observed changes in serum ASP levels after oral ingestion of a prostaglandin synthetase inhibitor (Etheridge *et al.*, in press) suggest that ASP is closely related to some aspect of prostaglandin physiology. For the past few years, prostaglandins have been increasingly implicated in many aspects of tumour development and growth (Editorial—*Lancet*, 1979; Jaffe, 1974). However, there is no evidence of an association between prostaglandins and breast cancer or leukaemias. The results of this study are of great interest in view of both the general elevation in serum ASP levels and, more important, the striking differences between the various types of leukaemia studied. At the present time, and until more is known about the exact biology of ASP, it is futile to speculate on the relationship between this protein and the neoplastic process; *e.g.* whether it is a primary product of the tumour cells, or the result of a secondary response to the tumour. Nevertheless, these preliminary observations should prompt further investigations into the pathological significance of raised serum levels of ASP in human neoplasms.

FIG. 2.—Serum ASP levels in pre-operative samples from patients with benign and malignant breast disease.

FIG. 3.—Correlation between ASP and CEA in patients with localized (○) and disseminated (●) breast cancer. Malignancy is strongly suspected with serum CEA levels equal to or above 30 µg/l.

We extend our thanks to Dr J. S. Malpas, Dr J. Habeshow for providing the serum samples from patients with leukaemia and to Mr W. P. Greening and Mr J. A. McKinna of the Breast Unit, Royal Marsden Hospital, for allowing us to study their patients.

### REFERENCES

Al-Awqati, M. A., Gordon, Y. B. & Chard, T. (1979*a*) Identification and purification of a new protein from water soluble extracts of human adrenal gland. *J. Endocrinol.*, **82**, 383.

Al-Awqati, M. A., Etheridge, R. J., Grudzinskas, J. G. & Chard, T. (1979*b*) Development of a radioimmunoassay for adrenal specific protein: Its measurement in biological fluids and tissues. *J. Endocrinol.*, **82**, 389.

Editorial (1979) Anti-inflammatory drugs and tumour growth. *Lancet*, i, **420**.

Etheridge, R. J., Al-Awqati, M. A., Gordon, Y. B., Grudzinskas, J. G. & Chard, T. (1980) The effect of flurbiprofen on serum levels of adrenal–seminal–pituitary protein (ASP). *Prostaglandins*. (In press.)

Jaffe, B. M. (1974) Prostaglandins and cancer: An update. *Prostaglandins*, **6**, 453.

Laurence, D. J., Stevens, U., Bettetheim, R. & 6 others (1972) Role of plasma CEA in diagnosis of gastrointestinal, mammary and bronchial carcinoma. *Br. Med. J.*, iii, 605.